# Medical students’ experience of emotions and success in neurological studies – What do they tell us?

**DOI:** 10.1186/s12909-017-0905-4

**Published:** 2017-04-04

**Authors:** Hanna Ansakorpi, Marja-Liisa Sumelahti, Raimo Kaasila

**Affiliations:** 1 Research Unit of Clinical Neuroscience, University of Oulu, B.O.X. 5000, 90014 Oulu, Finland; 2 Oulu University Hospital, B.O.X. 5000, 90014 Oulu, Finland; 3 School of Medicine, University of Tampere, 33014 Tampere, Finland; 4 Faculty of Education, University of Oulu, B.O.X. 2000, 90014 Oulu, Finland

**Keywords:** Neurology, Clinical teaching, Qualitative, Medical education, Undergraduate

## Abstract

**Background:**

There is a need to develop effective educational experience in neurology to improve the students’ skills in diagnosing and managing patients with neurological symptoms or disease. The aim of this study was to investigate the medical students’ attitudes and emotions towards neurology before and after the four week clinical course at two Finnish Universities in order to find elements to improve effective learning by decreasing the emotional stress in medical studies.

**Methods:**

In this two-stage study, 58 medical students participated in an internet survey with open-ended questions after completing a clinical neurology course.

In the content analysis of this survey 20 students (35%) were identified with negative anticipation towards neurology before undertaking the clinical neurology course. In the second phase of the study, the narrative analysis method was used to analyse the face-to-face interviews. Two of these interviews are described in this paper and represent cases who expressed negative emotions in both online survey and narrative interview.

**Results:**

According to the content analysis, the principal emotions that were experienced at the beginning of the clinical neurology course were insecurity about personal performance (*n* = 19, 95%) anxiety (*n* = 9, 45%) and fear (*n* = 6, 30%). During the course the combined negative emotions (insecurity, anxiety, and fear) decreased in 80% of students (16/20 cases), remained unchanged in 15% (3/20) and could not be evaluated in 1 (5%) case. The main reasons for the observed negative anticipation were the complexity of neurology and challenges in the interpretation of clinical findings. Based on content analysis and narratives, elements that were evaluated as the most significant contributors in reducing this included small group teaching with real patients, teachersʼ expertise and the increase in self-confidence.

**Conclusion:**

Teaching with appropriate didactic methodology and feedback, and plenty of practical training can improve effective learning in neurology. We suggest that the pedagogic competence of the clinical teacher influences a student’s motivation and proficiency and reduce stress in neurology-related learning tasks.

## Background

Medical school is an emotional experience for students. The influence of emotion on cognition is well recognized. Emotions affect learning of complex skills and knowledge and transferring information into new scenarios [1]. From the psychological and cognitive view it is believed that negative emotions narrow individuals’ momentary thought-action repertoires by calling forth specific action tendencies (e.g., attack, flee), whereas many positive emotions broaden individuals’ momentary thought-action repertoires, prompting them to pursue a wider range of thoughts and actions [2, 3].

In neurology, interviewing and examining the patient are crucial skills in both diagnosing and excluding a disease, as well as in the follow-up of disease progression. There is a need to develop effective educational experience in neurology as it has been reported that students experience neurology as a difficult topic, and some ascribe this to insufficient knowledge and poor teaching [4]. These experiences may have effect on number of medical students who continue to pursue careers in clinical neurosciences. Other factors that lead students to pursue or avoid careers in neurology have been related to emotional experiences during training [5–8].

Jozefowicz first represented a term ‘neurophobia’ describing it as a phenomenon where medical students are unable to apply knowledge of basic neuroscience to a clinical situation [5]. The ‘symptoms’ include intimidation, boredom and in some individuals, a cynical and nihilistic attitude towards neurological diseases in later career. A major ‘sign’ is inability to localise focal lesions in the nervous system. Although the term ‘neurophobia’ is informal, results of any such suggestion may in worst scenarios lead to real clinical consequences, and phenomena thus needs recognition, as many common neurological diseases among ageing populations worldwide are increasingly treated by primary health care physician [6, 9].

Previous studies suggested that integrating basic science and clinical neurology into medical school training in the form of group work and case-based exercises could reduce stress related to neurological studies [10]. However, solid qualitative evidence showing the effect of such interventions is lacking [11] nor have the causes for negative anticipation in neurological studies, which may differ from other issues in academic performance, such as procrastination, been extensively studied [12]. The amount of negative anticipation towards neuroscience that exists in Finland and factors that could reduce the incidence are not known. Evaluation is valuable as although being a common phenomenon [9], the degree of these attitudes and emotions may not be consistent between different countries or regions [7].

It is commonly acknowledged that the development of clinical reasoning skills is the most important goal of medical studies [9]. Clinical reasoning requires problem solving skills, which can be acquired by structured teaching [13, 14]. A good clinical teacher or an enthusiastic senior colleague may powerfully nurture learning by seeing the student’s point of view and applying pedagogic theories to teaching [14].

Teaching medical students how to perform a neurological examination is a challenge. It is not clear whether students are able to adopt hypothesis-based level of neurological examination, where clinical hypotheses that arises from the interview of a patient, steers the examination [15, 16]. Therefore, with regard to performing a neurological examination, some authors have concluded that it may be better to teach them more categorical screening type skills rather than how to formulate hypotheses [15, 17].

In Finland, all five universities use national learning objectives for medical students, although the curricula differ slightly between them. For example, in Oulu University, most neuroanatomy modules are incorporated into pre-clinical studies, and clinical neurology and clinical neuroanatomy are taught during the 4th year of study. In contrast, in Tampere University, neuroanatomy is integrated into clinical neurology during the 2nd to 3rd and 5th to 6th years of study. Approximately 135 students in Oulu University and 100 students in Tampere University attend these clinical courses each year. Clinical teaching is structured and takes place within small groups of 6–12 students, with the focus on clinical neuroanatomical knowledge. In practice, during a 4-week contact-teaching period, students examine patients with various neurological-related complaints, both in outpatient clinic and hospital ward settings. The training takes place within small groups, as well as individually, under the guidance of a clinical teacher. During this period, a thorough neurological examination is introduced but the focus is on the screening type of neurological examination, with some level of hypothesis-based examination in different clinical situations. The students receive constant, direct and individual feedback from teacher regarding their progress and performance. Students give both spontaneous oral feedback after each sessions as well as compulsory written feedback using a structured form and free word after the clinical course.

Clinical teachers are mainly responsible for teaching students how to conduct a neurological examination, although senior staff in the hospitals as well as health care centres are involved in some educational tasks besides their clinical work. Some Finnish universities require that all clinical teachers have a pedagogic education background, whereas others recommend it. At present, practically all clinical teachers of neurology in Finland have studied university pedagogy, at least to some extent.

This study was completed in two medical schools in Finland, Oulu and Tampere, during 2015. The aim of this study was to explore the types of negative emotions Finnish medical students have towards neurology and how to reduce these.

## Methods

### Phase 1: Online survey and content analysis

One hundred and thirty-five students (Oulu: *n* = 95; Tampere: *n* = 40), were invited to take part in an anonymous survey, using an online survey tool (Webropol) after completing their clinical neurology course. No ethical approval was required by the Regional Ethics Committee of Northern Ostrobothnia Hospital District. A written consent was obtained from the study subjects before entering the study. The purpose of the first phase online survey was to study attitudes and experiences widely.

The open-ended questions in the questionnaire for the assessment of experiences and attitudes in neurological studies were:

- ‘What were your experiences of your neurological studies compared to those of other specialties?’ ‘Please elaborate on the reason for these’.
- ‘Please describe your experience of performing a neurological examination on a patient. What was a) the most difficult aspect and b) the most interesting aspect?’
- ‘Please state how capable you feel about a) conducting a neurological examination of a patient and b) interpreting the neurological findings of the examination’.
- ‘Has your attitude towards neurological patient examinations changed since you completed the clinical course and, if so, how?’

### Content analysis

Open questions in the first phase of the study explored students’ experiences. Both positive and negative experiences were screened. Descriptions of emotions were picked out and classified as negative in case of e.g. anger, anxiety, fear, and positive if they conveyed optimism, contentment, and happiness. In categorization we followed the widely used terms in Emotion Report Forms [18]. We focused on negative experiences and in the narrative phase of the study explored this further among the students that expressed them.

We used content analysis to explore the emotions and to examine trends and patterns in attitudes [19, 20]. The change of the nature of emotions before and after the clinical neurological course was evaluated. Emergent coding of categories expressed negative/positive emotion took place after a preliminary examination of the data. To control for reliability and validity, an intra- and inter-rater assessment was done. First, H.A. and M.S. independently reviewed the data and searched for descriptions related to emotions. The results were then compared, and differences were reconciled by consensus. After reconciliation of the data, a consolidated checklist was drawn up, and H.A. and M.S. applied the coding. The reliability of the coding was checked by comparing the results, which revealed a good level of reproducibility. To determine the intra-rate stability, a second round of coding took place 6 months later. The results showed good reliability.

### Phase 2: Narrative interview

In the narrative phase of the study, an invitation to attend a personal interview was sent to the initial online survey responders (*n* = 58). Of these, 11 (21%) students were willing to relinquish their anonymity and participate in face-to-face interviews.

### Narrative method

The narrative analysis is commonly used in social sciences [21–23]. The basic premise of narrative inquiry is that people make sense of themselves and their world by telling stories [21]. The narrative interviews with the medical students lasted 20–30 min and were conducted as described previously [22, 23]. Confidentiality was assured, and a relationship between the interviewer and interviewees was established. The interview consisted of asking the students to tell stories related to their experiences of the clinical neurology course, using open-ended prompts.

For this study, we systematically selected two cases, Tomi and Petri, for examination. We used a critical case strategy by selecting medical students who would contribute the most towards understanding students’ experience of emotions and success in neurological studies. The cases selected made a point clearly, were particularly information rich [24] and expressed themselves vividly [25].

The literature presents various approaches to conducting narrative analysis [23, 26, 27]. The narrative inquiry that we applied here involved emplotment [26, 27]. Like Ricoeur [28], we take the view that the plot brings together goals, causes and chance within the temporal unity of a whole action. In particular, when emplotting Tomi’s and Petri’s narratives our goal was to explicate how their experiences in participating the clinical neurology course influenced their emotions towards neurology. The emplotment began by specifying the outcomes in Tomi’s and Petri’s narratives. The main outcome was considered a positive change in students´ emotions towards neurology after the course. Then, with reference to the data, the interviewee was asked about how the change happened, and we began seeking clues from the interviews that seemed to ‘explain’ the change and to reduce negative emotions towards neurology. When constructing the final version of the students’ case descriptions, we arranged the data elements chronologically. To give a voice to the students we utilized many direct quotes from their talk. We also analysed the way the students talked, especially central expressions that they used when they talked about their experiences during the clinical neurology course, because it helped us to understand their purposes and actions. At the end of both case descriptions, we present a short summary of the cases where we explicate how the process of change happened, and what factors seemed to facilitate the change. We also connect the students’ narratives to the broader theoretical framework that was used to interpret the narrative.

## Results

### Internet survey

Of 135 students, 58 (35 females and 23 males, 43%) responded to the initial online survey. Of these, 43 (74%) were from Oulu University, and 15 (26%) were from Tampere University. All the 58 questionnaires were completed correctly, and all the data were therefore usable.

### Content analysis

At the beginning of the clinical neurology course, 20 (34%) of the responders (14 [33%] from Oulu University and 6 [40%] from Tampere University; 12 females and 8 males) conveyed negative emotions.

**Fig. 1:**
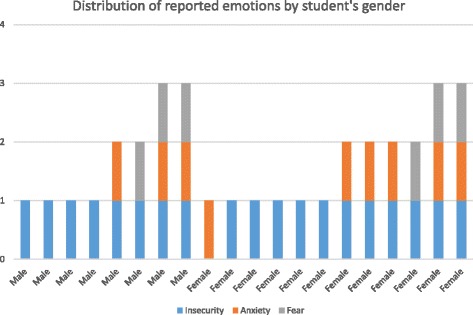
Distribution of reported emotions before the clinical neurology course, according to the student’s gender

The analysis of the data revealed that emotions could be categorised to: insecurity about personal performance (*n* = 19; 95%), anxiety (*n* = 9; 45%) and fear (*n* = 6; 30%). In half of the cases (*N* = 10), both sexes reported more than one emotion. All three emotions (insecurity, anxiety and fear) were more common among males (2/8; 25%) than females (2/12; 17%). The distribution of emotions experienced before the course is illustrated in Fig. 1.

**Fig. 2:**
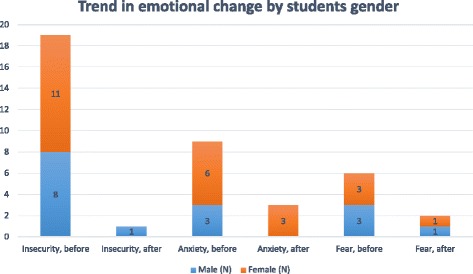
Trends in the change of emotions among medical students before and after the course in clinical neurological studies

During the course the combined negative emotions (insecurity, anxiety, fear) decreased in the majority of students (16/20, 80%), remained unchanged in some students (3/20, 15%) and could not be evaluated in one case (5%). Insecurity observed in 19 cases decreased significantly (18/19, 95%), but remained unchanged in one case (5%). Anxiety and fear decreased in most students (6/9, 67% anxiety; 4/6, 67% fear) and remained unchanged in one third of cases (3/9, 33% anxiety; 2/6, 33% fear). The histogram in Fig. 2 illustrates the number of reports and the trends in change.

Students’ open ended answers unanimously pointed out that improvements in examination skills mainly explained the decrease in negative emotions. The students considered structured neurological examination instruction and practice beneficial. Most of the students viewed developing an understanding of how to interpret the neurological findings as the main reason for the decrease in their negative emotions.

### Narratives

Total of 11 (21%) of the 58 online survey responders, were willing to relinquish their anonymity and participate in 20–30 min face-to-face interviews.

Below, we present the experiences of Tomi and Petri of their clinical neurology studies and the emotions they experienced.

**Tomi’s case:**

Before the clinical neurological course, which took place during the 5th year, Tomi said: *‘I was anxious about having to deal with difficult neurological diseases, such as Parkinson’s disease and stroke. During the study break, I worked on a primary healthcare ward and met a patient with end-state Parkinson’s disease who had swallowing difficulties. I found it difficult to make medical decisions. I was not ready to take responsibility for such a severe case’.*

Above Tomi described well his anxious feelings towards neurology. It seems that his challenging experiences in working on a primary healthcare ward even increased his anxiety. The reason for his anxiety were ‘*difficult neurological diseases, such as Parkinson’s disease and stroke’.* Tomi assumed that both acute and chronic neurological diseases were severe. This gave rise to anxiety about his ability to manage neurology patients. He concluded: *‘My skills are not at an adequate level to treat difficult neurological cases’.*

Tomi further noted:

*‘Before starting the clinical course, we had all taken part in tutored practice on neurological examination, and I had practiced it also at work. I was able to do “the tricks”, but I didn’t understand their clinical significance’.*

Here Tomi’s expression ‘*I was able to do the tricks’* shows well that his learning had been on a superficial level, and deeper learning with understanding was missing.

*‘My motivation to learn was good, due to primary experience. During the course, I recognised the learning objectives better. Even if I did not have the chance to study all the patients myself, it was helpful to observe the examination of patients with different sorts of neurological complaints’.*

He was aware that he had forgotten what he had learned earlier about anatomical nervous system structures and basic clinical neurology.

Tomi also noted:

*‘Pieces finally clicked into their place in the neurological examination’.*

During the clinical studies, Tomi felt comfortable examining a patient when the teacher was present, as the teacher guided him through the structured neurological examination, as illustrated below:

*‘It was important to see how an examination should be done and to observe abnormal findings, such as a clonic reflex’.*

Although Tomi felt that it was important to be able to examine several patients, he also felt that the quality of the teaching was more important than the quantity of the patients observed. Furthermore, he remarked that a friendly and open-minded atmosphere made it easier to ask questions and discuss the patient cases freely.

During the rounds at the university hospital, he met tertiary care patients with rare and severe neurological conditions. He met these patients without having a comprehensive knowledge on their diseases, although he had read their case records. He recounted the following:

*‘Things proceeded too fast and the information was way too complex! For example, during the clinical rounds, one specialist immediately engaged in a complex discussion of the clinical problem, including a huge amount of detailed information. In that situation, his questions were difficult to comprehend, and I was unable to come up with answers. As a result, I felt stupid’.*

Tomi further elaborated on the problems experienced during the clinical rounds, as follows: *‘The specialist did not seem to understand our level of knowledge or remember what it had felt like to be a student. It would have been helpful if the senior doctor had clarified his decision-making process. The decision-making seemed to be based on intuition, and he did not explain the process that led to the decision. As a result, I felt that I learned less than I should have in these situations. However, during other clinical rounds, an esteemed senior doctor talked casually before the rounds, and this created a relaxed atmosphere’.*

*‘I think that it is important, particularly during rounds, to create a welcoming atmosphere, where even stupid questions are allowed and where a student can ask for clarification if he/she does not understand the question. To improve learning, I feel it is important to dare to be stupid!’*

Here Tomi expresses his frustration on the experienced poor teaching skills of a senior doctor and comments on the importance of pleasant atmosphere during teaching sessions.

‘*In the outpatient clinic, if I do not have a clear hypothesis at the outset, I just start to take a patient history and then examine the patient. Today, after I have taken a complete patient history and examined the patient, I feel I can arrive at a working diagnosis. I also feel more confident about consulting specialists’.*

The data above shows that Tomi’s self-confidence is nowadays much better than it was in the in the outpatient clinic.

Since completing his clinical neurology studies, Tomi has met several neurological patients at different clinics, and he feels at ease with the examinations. At the emergency clinic, he has also been able to incorporate hypothesis-based reasoning into the neurological examination.

*‘Concerning the patient examination, I now understand how to diagnose signs and symptoms at the neuroanatomical level. Since completing the course, I have seen a number of patients, most of whom have subacute cerebral symptoms. In all cases, I have been able to figure out the level of neuroanatomical symptoms and signs quite quickly’.*

*‘If I have studied the case carefully, I am better able to discuss the problem with the senior consultant. In many cases, I have identified the source of the problem. I feel much more confident when examining different patients, and I am not afraid anymore. Most of the time, I am able to consult the right specialist, and I reach the correct diagnosis’.*

### Summary of Tomi’s case

Tomi had earlier experience of dealing with patients with neurological diseases on a primary health care ward. Prior to starting his clinical neurology studies, he felt that his pre-clinical preparation was sufficient. He also had hands-on experience of conducting neurological examination. He felt that this experience provided a solid foundations for the clinical neurological course. He felt demotivated by the busy atmosphere during the rounds and rare tertiary care patients. He did not understand the level of knowledge that senior specialists expected him to have. He felt that the communication between the students and senior doctors was inadequate. His experiences of busy rounds and senior doctors made him feel inferior. He did not learn well in these situations. He also felt that he should have received more tuition in diagnostic reasoning skills. The main positive elements of his learning experience were structured teaching with varying teaching sessions. The increase in self-confidence decreased Tomi’s anxious feelings. The negative elements were related to emotional experiences during the course.

### Petri’s case

Petri had studied neuroanatomy (2nd year) and neurological diseases (3rd year). He remarked:

*‘Neuroanatomy did not interest me… I knew that it was important, but I did not study it that well, and I was not interested either. However, this made the following courses difficult’.*

The data above shows that Petri’s motivation of learning neuroanatomy was quite low although he seemed to appreciate the topic.

Petri further noted:

*‘The neurological diseases I knew about were gloomy and depressing. They are progressive, and there is no cure for them. They frightened me. Some members of my family had multiple sclerosis and amyotrophic lateral sclerosis, so I had personal experience of the diseases, and I felt anxious. At work, I had met stroke patients. As I had no neurology training, it found my dealings with them difficult, I felt I should have paid more attention to neurology studies’.*

*‘Neurological studies took a lot of time. I felt anxious and fearful because I did not have enough knowledge to do this course!’*

Above Petri used many string emotional expressions like “they frightened me” and “I felt anxious” that showed well that he had negative anticipation already before the studies. The main reason for this seemed to be the fact that he had “personal experience of the diseases” because some of his family members had had neurological diseases. He also felt that he did not have enough neurology training to deal with stroke patients.

He had to study a lot to learn neuroanatomical basics, in addition to clinical practice, which was time consuming, as noted below:

*‘I studied a lot. The integration of basic neuroscience and clinical examinations made me feel more confident. We met several patients and practiced neurological examinations so many times that I did not have to think about the mechanical performance and therefore had time to engage in clinical reasoning’.*

The data above shows that Petri’s self-confidence was improved through the integration of basic neuroscience and clinical examinations. This was a turning point of his narrative.

During the rounds, he found dealing with tertiary care patients (e.g. those with refractory epilepsy) confusing because of the complexity of symptomatology and treatment options. He also felt that managing acute stroke was more difficult and demanding than managing other neurological diseases and that his emotional stress level was greater when he met patients with progressive neurological diseases. However, he realized that there were many common neurological symptoms and disorders and that he should focus mainly on them.

*‘I felt uncomfortable when meeting patients who had been told they had a rare, fatal disease when their symptoms had at first seemed benign. This interfered with my diagnostic reasoning’.*

Further, he emphasised: *‘Neurological diseases, they ARE just more complex than other diseases!’*

Although Petri’s self-confidence increased with the developing skills in neurology, anxious feelings did not disappear.

After the clinical neurological course, while working during the summer break, he met several patients with neurological symptoms*.* Strokes made him feel anxious, as they are so common, and there is a lot to study, as shown below:

*‘Some of the symptoms were difficult to define. It helped when I performed a thorough neurological examination. However, I had to keep an open mind. The differential diagnosis: that was difficult. Still, I felt I had sufficient knowledge on the most common types of neurological diseases to deal with the cases’.* He added: *‘I felt good at work and liked neurology. I could even diagnose a cluster headache!’*

For students dealing with negative emotions towards neurology, Petri says they need to realise that it will take time to amass the knowledge needed to understand clinical neurology.

‘*Neurology is such a wide and difficult discipline, and I revere it. To learn, you have to study hard, more so than with other specialties. However, I know now that it can be done!’*

Here Petri’s talk is decisive; he has found a resolution for overcoming his challenges in “study hard”. Petri knows what he wants for the future and therefore uses utterances such as “*I know now that it can be done!’*

### Summary of Petri’s case

Petri was not interested in neurology before the clinical course. He had neglected pre-clinical neurological studies because they caused him anxiety. Personal experience of neurological disease in his family had given rise to feelings of fear. During the course, structured teaching, practice and a good atmosphere motivated him to learn. Extensive studying further helped. Active participation in teaching sessions and self-directed learning increased his confidence. Poor preparedness on his part for clinical neurology and encounters with frightful diseases decreased his motivation. Today, Petri is confident about dealing with acute neurological patients but continues to feel emotional stress in relation to neurological diseases. Thus, he has not considered neurology as his future specialty.

## Discussion

In this study, the complexity of neurology and the interpretation of clinical findings were the main causes of negative emotions among the students. Structured teaching effectively reduced these emotions towards neurology, whereas non-structured teaching seemed to increase such emotions. In structured medical teaching, the learning objectives are clear and appropriate, and teachers’ didactic methods are suitable for small groups, with supervision and immediate feedback. The teaching focuses on common neurological symptoms and diseases and proceeds from the signs and symptoms to a diagnosis. In Finland, students are expected to acquire the skills needed to work in general practice during their clinical neurology studies. The findings of the present study provide further evidence that the integration of basic neuroscience, anatomy and clinical neurology into training improves problem solving in neurology [29–32].

The students in this study were in the final stages of their studies, and they were about to enter their working lives, with their current attitudes and experiences. We believe that this was an appropriate time to evaluate their learning experiences and self-assessment of their clinical neurological skills. In the voluntary internet survey, a 43% compliance rate was reached, and those who participated returned completed questionnaires, all of which were included in the study. Although the narrative examples are those of two male students, their attitudes were representative of those of the other students with negative anticipation in the cohort and logical generalizations are still possible in the sense of "if it happens there, it can happen anywhere" [24].

The findings showed that students’ preconceptions can change. The narrative part of the study demonstrated that Tomi and Petri did well in their neurological studies and that they are gifted students. Despite this, they had negative emotions towards neurology and their ability to learn it. The negative emotional experiences arose from past exposure to neurology. In Tomi’s case, this was a patient with end-stage Parkinson’s disease, and in Petri’s case, it was severe neurological diseases in the family. Tomi also felt that poor communication with the instructors and poor teaching skills among senior doctors increased his anxiety and affected his self-esteem. In contrast, Petri had high demands towards his own level of knowledge, which caused feelings of inadequacy in neurology as well. In both students, these emotions disappeared during the clinical course. Not only Tomi and Petri but also the other participants reported that structured teaching and increased exposure to patients were the most helpful methods to enhance learning in neurology and to overcome the emotional obstacles to learning. It also seemed that with the developing clinical skills in neurology the self-confidence increased, which had a positive effect on the anxious emotions of the students. These two narratives represent cases who expressed negative emotions in both online survey and narrative interview. In both cases negative anticipation decreased during the course. We believe that they are representative examples in the cohort and also represent the substance relevant to our study question.

The method used in this study combined qualitative and quantitative methods. The methodology used in this study is new in the field of neurological pedagogic research [33]. A content analysis is considered a useful tool for examining trends and patterns and provides a basis for monitoring shifts in attitudes [19, 20] and it is also a powerful data-reduction technique. The narrative analysis is a commonly used qualitative method in social sciences [21–23]. The basic premise of narrative inquiry is that people make sense of themselves and their world by telling stories [21].

By utilising these methodologies, we believed that we could achieve a broader understanding of students’ perceptions of learning neurology. The use of open-ended rather than closed-ended questions in the online survey allowed the students to describe their experiences in their own words. The narrative method deepened the descriptions and helped us to better understand the processes underlying the expressed emotions. The small number of students in this study also directed the choice of methodology.

As noted elsewhere, teaching methods may need to be revised to improve the integration of basic science knowledge and clinical neurology into medical training, for example, including virtual cases and group patient meetings [29, 34, 35]. However, it also needs to be recognised that learning clinical neurology is not only a cognitive but also an emotional process, which should be consciously supported by teachers [36]. We believe that such support is the best way to meet the individual learning trajectories of medical students. Furthermore, as also stated previously [10, 30, 37], according to the opinion of the students in this study, pragmatic training with actual patients under supervision leads to the best possible results.

As noted in an earlier study, the way in which negative emotions influence evolving professional self-esteem, in this case, that of medical students, is unclear [38]. However, we can speculate that if there is a connection, it is adverse. In the present study, most types of negative emotions were already present in the content analysis. However, content analysis is best suited to small cohorts [19, 20]. To study a similar phenomenon in medical students in general, there is a need for validated methods for the assessment that would suit analysing larger study samples.

Based on our results, neurology seems to make also Finnish medical students nervous. In this study, the concept of ‘neurophobia’, a phenomenon originally described by Josefowicz [4] as not totally serious, became more precise, as more than fear, feelings of anxiety and insecurity were observed, in addition to the preconception that neurology is a difficult discipline in medicine. A neurological examination is certainly akin to a 3D jigsaw puzzle, where a diagnosis is reached by the clinical problem solving that is based on neuroanatomical knowledge, as also corroborated by others [14]. Therefore, elaborating on the clinical thinking underlying individual cases may help to convert abstract concepts into concrete reasoning.

Limitations of our study concern the small sample size, limiting inferences of the analyses. Another limitation concerns the self-reporting, as online survey and interviews took place only after the neurology course. Self-reports of current emotional experiences are likely to be more valid than are self-reports of emotions made somewhat distant in time from the relevant experience [39]. Results observed in this study are aimed to be evaluated in a larger student cohort, where currently experienced emotions are assessed with suitable questionnaires for stress, anxiety and goal orientation [40].

In the research literature, the concept of phobia has often been described as an irrational fear of specific objects that is not under voluntary control and that often leads to the avoidance of the phobic situation [41]. For example, Tobias described ‘mathphobia’ as an irrational fear of mathematics [42]. Cemen defined ‘mathematics anxiety’ as a state of discomfort that occurred in response to situations involving mathematical tasks, which were perceived as undermining the person’s self-confidence [43]. Based on the findings of the present study, we suggest that ‘neurophobia’ can also be manifested as ‘neuroanxiety’ because the negative emotions that the students had were not irrational. The findings in our study using content analysis complemented by narrative methodology however showed that students’ preconceptions can change, enabling intervention with competent teaching and emotional support.

## Conclusion

Although the perspective was to study negative repertoires, our interest was to explore which factors decreased them and brought up the positive action tendencies. We observed these positive tendencies and believe that teacher’s awareness of them broaden the scope of attention and thought action repertoires during the neurological studies [44]. Emotions may influence medical education in several ways that need further exploring. Our observations are in accordance with a concept that learning should not be treated simply as a rational, mechanistic process because emotional conditions are shown to affect the performance [1]. Validated methodology to study the current emotions in larger student cohorts would help to adjust teaching to meet also the attitudes and emotional needs of medical students.
